# Identification of Characteristic Bioactive Compounds in Silkie Chickens, Their Effects on Meat Quality, and Their Gene Regulatory Network

**DOI:** 10.3390/foods13060969

**Published:** 2024-03-21

**Authors:** Xinting Yang, Chaohua Tang, Bowen Ma, Qingyu Zhao, Yaxiong Jia, Qingshi Meng, Yuchang Qin, Junmin Zhang

**Affiliations:** State Key Laboratory of Animal Nutrition and Feeding, Institute of Animal Sciences, Chinese Academy of Agricultural Sciences, Beijing 100193, China; yangxinting0704@163.com (X.Y.); tangchaohua@caas.cn (C.T.); mbwxumu@163.com (B.M.); zhaoqingyu@caas.cn (Q.Z.); jiayaxiong@caas.cn (Y.J.); mengqingshi@caas.cn (Q.M.); zhangjunmin@caas.cn (J.Z.)

**Keywords:** silkie chicken, characteristic metabolite, characteristic lipid, meat quality, volatile flavor compound, gene regulatory network

## Abstract

Silkie chicken, an important chicken breed with high medicinal and nutritional value, has a long history of being used as a dietary supplement in China. However, the compounds with health-promoting effects in Silkie chickens remain unclear. In the present study, we conducted a comprehensive analysis of metabolic and lipidomic profiles to identify the characteristic bioactive compounds in Silkie chickens, using a common chicken breed as control. The results showed that the levels of 13 metabolites including estradiol, four lipid subclasses including cardiolipin (CL), eight lipid molecules, and three fatty acids including docosahexaenoic acid (C22:6) were significantly increased in Silkie chickens, which have physiological activities such as resisting chronic diseases and improving cognition. These characteristic bioactive compounds have effects on meat quality characteristics, including improving its water-holding capacity and umami taste and increasing the content of aromatic compounds and phenols. The differentially expressed genes (DEGs) between the two chicken breeds revealed the regulatory network for these characteristic bioactive compounds. Fifteen DEGs, including *HSD17B1*, are involved in the synthesis of characteristic metabolites. Eleven DEGs, including *ELOVL2*, were involved in the synthesis and transport of characteristic lipids and fatty acids. In summary, we identified characteristic bioactive compounds in Silkie chickens, and analyzed their effects on meat quality characteristics. This study provided important insight into Silkie chicken meat as a functional food.

## 1. Introduction

“Silkie chicken” is a unique chicken breed originating from Taihe County in Jiangxi Province, China. It has snowy white feathers and black skin, muscles, and bones. The history of consuming meat from Silkie chickens is long and profound. It is recorded in Chapter 154 of *The Travels of Marco Polo* from the late 13th century. According to the Ming Dynasty medical book *Shou Shi Bao Yuan*, after removing feathers, internal organs, and subcutaneous fat, fresh male Silkie chicken is cooked together with 12 traditional Chinese medicinal materials. The cooked meat and medicinal materials are minced and dried into powder together, and rice wine is added to make “Black Chicken Pills”, which are used to nourish blood and to treat menstrual irregularities, and postpartum disease. The fresh skin, muscle, and bone of Silkie chickens are all recorded as traditional Chinese medicinal materials in the *Pharmacopoeia of the people’s Republic of China* (2020) (https://chp.health-china.com/, accessed on 22 January 2024). Nowadays, the meat from Silkie chickens is considered a functional food product with high nutritional value, and the elderly and women are more inclined to eat Silkie chicken meat to nourish their body.

Although the health-promoting effects of Silkie chicken meat have been recognized for a long time [1,2], the key bioactive components remain unclear. Existing studies have preliminarily analyzed the bioactive components with high nutritional value in Silkie chicken meat. A notable characteristic of Silkie chicken meat is the distribution of melanin [3], which has health-promoting effects such as nourishing blood, hypoglycemic effects, and antioxidant activity [4]. With respect to water-soluble metabolites, the carnosine content in the breast muscle of Silkie chickens is significantly higher than that in the breast muscle of White Plymouth Rock [5]. With respect to fatty acids, the contents of polyunsaturated fatty acids, arachidonic acid (20:4n − 6), eicosapentaenoic acid (20:5n − 3, EPA), docosapentaenoic acid (22:5n − 3, DPA), and docosahexaenoic acid (C22:6n − 3, DHA) in the breast muscle of Silkie chickens are significantly higher than those in the breast muscle of Cobb chickens [6], crossbred Silkie chickens [7], Lingnan yellow chickens, and Chongren chickens [8]. However, the above studies only compared the contents of several specific metabolites and fatty acids in the breast muscles of Silkie chickens and other chicken breeds.

The meat from Silkie chickens is firm and delicious. A previous study showed that compared with Korean local chicken, meat color L*, a*, and b* values of breast muscle in Silkie chickens were lower and the pH value and water-holding capacity were higher [9]. A study on volatile flavor compounds showed that the content of aldehydes and esters in the breast muscle of Silkie chickens was significantly higher than that in the breast muscle of Cobb chickens [6]. Small-molecule metabolites and lipids are closely related to meat quality characteristics. Energy metabolism, fatty acid synthesis, and lipid accumulation in skeletal muscle regulate and determine meat quality traits in a coordinative manner, including color, pH value, water-holding capacity, intramuscular fat content, and tenderness [10]. Water-soluble metabolites and lipids are important flavor compounds and form volatiles through the Maillard reaction and lipid oxidation, respectively, which determine flavor quality [11]. However, there is currently a lack of research on the relationship between meat quality characteristics and metabolites in breast muscle of Silkie chickens.

Age has a significant effect on the contents of metabolites and lipids in muscle [12]. The marketing age of commercial broilers is generally 6 weeks. However, many Chinese local chicken breeds grow slowly, with a marketing age of more than 16 weeks. These local chickens are delicious and nutritious. Consumers usually cook the meat in purified water to retain its original taste and nutrients. Silkie chicken and Wuding chicken are two such famous local breeds, and have the same growth rate and marketing age (24 weeks). Wuding chicken is a high-quality local chicken breed with tender and delicious meat that has a broad consumer market in southern China. However, consumers eat the meat of Wuding chicken as ordinary food, while the elderly and women prefer the meat of Silkie chickens as functional food to nourish their body. Therefore, in the present study, we conducted metabolomic and lipidomic analyses to comprehensively identify the characteristic bioactive components with high nutritional value in breast muscle of Silkie chickens, and analyzed the effects of these characteristic components on meat quality characteristics.

## 2. Materials and Methods

### 2.1. Ethics Statements

The Animal Care and Use Committee of the Institute of Animal Sciences (IAS) at the Chinese Academy of Agricultural Sciences (CAAS) granted approval for all animal procedures in this study (IAS2023-108). Procedures and methods for the experiment adhered to authorized guidelines, ensuring the welfare of the animals involved.

### 2.2. Birds and Breast Muscle Sample Collection

One hundred male Silkie chickens and 100 male Wuding chickens were raised from 1 day old at the experimental sites of the IAS within the CAAS located in Beijing, China. These chickens were housed together in the same facility, and they were provided with the same commercial feed and drinking water, allowing them to feed and drink freely. When they reached 24 weeks of age, 30 male Silkie chickens and 30 male Wuding chickens were randomly selected and euthanized by carotid artery bleeding after a 12 h fasting period during the night. The breast muscles on each side were promptly isolated, and visible fat as well as connective tissue were eliminated. The left-side breast muscle was quickly frozen in liquid nitrogen and preserved at −80 °C. This preservation was performed for the analysis of metabolome, lipidome, and melanin contents, electronic tongue analysis, volatile compounds and transcriptome. The breast muscle on the right side was kept at 4 °C for 24 h to assess pH value, meat color, water-holding capacity and shear force.

### 2.3. Nutritional Value

#### 2.3.1. Metabolomic Analysis

The 50 mg of breast muscle sample was placed in 500 μL of prechilled 80% (*v*/*v*) HPLC-grade methanol and homogenized on dry ice. The samples were stored overnight at −80 °C. The samples were centrifuged at 14,000× *g* at 4 °C for 20 min in a refrigerated centrifuge to precipitate proteins. An equivalent volume of supernatant was moved to a new Eppendorf tube, and the samples were dried using a Speedvac. Subsequently, the samples were stored at –80 °C.

All procedures were conducted by modifying the established methods [13]. Positive mode: a BEH Amide column (2.1 mm × 100 mm, Waters, Milford, MA, USA). Mobile phase A: 5% water, 95% acetonitrile, 10 mM ammonium formate. Mobile phase B: 50% water, 50% acetonitrile, 10 mM ammonium formate. Linear gradient: 0–15 min, 1–99% B; 15–17 min, 99% B; 17–20 min, 1% B. Negative mode: a BEH C18 column (2.1 mm × 100 mm, Waters). Mobile phase A: HPLC-grade water, 5 mM ammonium bicarbonate, pH 8.0. Mobile phase B: HPLC-grade acetonitrile, 5 mM ammonium bicarbonate, pH 8.0. Linear gradient: 0–12 min, 1–99% B; 12–17 min, 99% B; 17–20 min, 1% B.

The analysis was performed on a Q Exactive Orbitrap MS (Thermo Fisher, Waltham, MA, USA) with the following parameters [14]. The data were analyzed using tracefinder (Thermo), and metabolites were identified and quantified by scanning a self-constructed database.

#### 2.3.2. Lipidomic Analysis

The 40 mg of breast muscle sample was placed in 300 μL of methanol and homogenized using a homogenizer. The sample was vortexed after adding 1 mL MTBE. The sample was subjected to liquid–liquid extraction by adding 250 µL of aqueous solution. The sample was vortexed for 1 min and allowed to sit for 20 min, and this process was repeated three times. The sample was centrifuged at 8000 rpm for 15 min. An equal volume of the upper organic phase was transferred to a new glass tube. The sample was dried under nitrogen to a clear film. The sample was preserved in a freezer at −80 °C.

According to previous studies with modifications [13,15], a Cortecs C18 column (2.1 mm × 100 mm; Waters) was employed in both positive and negative modes. Mobile phase A: HPLC-grade acetonitrile containing 10 mM ammonium acetate, HPLC-grade water (60:40, *v*/*v*). Mobile phase B: HPLC-grade isopropanol, HPLC-grade acetonitrile (90:10, *v*/*v*). The gradient: 0–23 min, 30–98% B; 23–30 min, 98% B; 30–35 min, 98–30% B.

The data acquisition was performed using a Q Exactive Orbitrap MS (Thermo) coupled with a UHPLC system Ultimate 3000 (Thermo). The mass spectrometry conditions were based on previous studies [14]. The lipids were identified and quantified using LipidSearch software 4.1.30 (Thermo, CA).

#### 2.3.3. Melanin Content Measurement

Melanin content was indirectly quantified using 1*H*-pyrrole-2,3-dicarboxylic acid (PDCA) and 1*H*-pyrrole-2,3,5-tricarboxylic acid (PTCA), which are the hydrolysates of melanin under alkaline hydrogen peroxide oxidation condition [16]. We accurately weighed 0.1 g of breast muscle sample, and added 0.5 mL of 30% hydrogen peroxide and 0.5 mL of 2 M ammonium hydroxide. We kept the meat sample soaked under the liquid surface, and stored it at 30 °C for 8 h. We then added 400 µL of 11.3% ammonium sulfite solution to the centrifuge tube. Subsequently, to modify the acidity for solid-phase extraction (SPE) column clean-up (Bond Elut SAX, 500 mg, 3 mL), 400 µL of 4 M acetic acid was introduced. Conditioning of the columns involved the use of 1 mL methanol followed by 1 mL of ultrapure water. The entire sample was subsequently introduced into the SPE column, allowing it to flow through the column using gravity. We added 3 mL of water and methanol in sequence for the washing process. The SPE column was then thoroughly dried using a vacuum pump. Elution was carried out with two portions of 1 mL of a 10% (*v*/*v*) triethylamine in methanol solution. The samples were dried under nitrogen gas. We then redissolved the dried sample in 1 mL of methanol containing 0.5% formic acid.

The reconstituted sample underwent analysis, with us utilizing an LC–ESI–MS/MS system (QTRAP 6500, Sciex, Framingham, MA, USA) equipped with an ESI Turbo ionspray interface. The liquid chromatography column: Agilent (Santa Clara, CA, USA) ZORBAX C18 (3 mm × 150 mm). Mobile phase A: water containing 0.1% acetic acid. Mobile phase B: acetonitrile containing 0.1% acetic acid. Gradient program: 2% B, 0 min; 2% B, 1 min; 20% B, 5.6 min; 100% B, 7.0 min; 100% B, 9.4 min; 2% B, 9.6 min; 2% B, 12.0 min. Flow rate: 0.35 mL/min. Column temperature: 45 °C. The mass spectrometer (MS) parameters were set as follows. Source temperature: 500 °C. Negative ion spray voltage: (−) 4500 V. Gas I: 50 psi. Gas II: 50 psi. Curtain gas: 35 psi. Collision gas: medium. The multiple reaction monitoring transitions were PTCA 198 → 154 (quantifier) and 198.13 → 110.19 (qualifier), PDCA 154.13 → 110.13 (quantifier) and 154.13 → 66.15 (qualifier).

### 2.4. Sensory Characteristics

#### 2.4.1. pH Value and Meat Color

The right-side breast muscle, which was stored at 4 °C for 24 h after slaughter, was placed flat on a white plastic tray. A calibrated pH meter (HI99163; HANNA, Woonsocket, RI, USA) glass electrode was attached to a metal knife edge and inserted directly into the breast muscle. Once the reading stabilized, it was recorded as the 24 h pH value.

A calibrated CR-400 Chroma meter (Konica Minolta, Tokyo, Japan) lens was positioned vertically above the muscle surface, with the lens in close contact with the muscle surface. The meat color L*, a* and b* were measured and recorded.

#### 2.4.2. Water-Holding Capacity

Water-holding capacity includes pressing loss and cooking loss.

Pressing loss was measured following the previous procedure [17]. A meat column was obtained from each fresh sample stored at 4 °C for 24 h using a sampler. The meat column was weighed using an electronic balance and recorded as *m*_1_. It was then wrapped in a double layer of gauze and further enveloped in 16 layers of qualitative filter paper. A force of 35 kg was applied using an infinite compression device for a duration of 5 min. After removing the gauze and filter paper, the meat column was reweighed and recorded as *m*_2_. Pressing loss (%) = (*m*_1_ − *m*_2_)/*m*_1_ × 100%.

Using a sharp knife, the fresh breast muscle was cut along the direction of muscle fibers to create a meat piece measuring 3.0 cm in thickness, 5.0 cm in length and 5.0 cm in width. The meat piece was weighed using an electronic balance and recorded as *m*_1_. The meat piece was then placed in a plastic cooking bag, and the bag was sealed with a clip. The meat piece, enclosed in the plastic cooking bag, underwent immersion in the water bath held at a stable temperature of 80 °C. This continued until the internal temperature of the meat sample achieved 70 °C. After removal, it was cooled for 30 min, and then stored it in a 4 °C refrigerator for 12 h. After letting it reach room temperature for 30 min, the surface moisture from the meat piece was absorbed using qualitative filter paper, and its weight was recorded as *m*_2_. Cooking loss (%) = (*m*_1_ − *m*_2_)/*m*_1_ × 100%.

#### 2.4.3. Shear Force

The cooked meat piece was further divided into six 1 cm × 1 cm × 2 cm meat strips along the direction of muscle fibers, with us taking care to avoid visible connective tissues, blood vessels or other defects. These meat strips were used for shear force measurement. The measurement of shear force was conducted using the TA-XT plus Texture Analyser (Stable Micro Systems, London, UK). This was achieved by employing the Warner–Bratzler shear force blade mode with a wedge-shaped probe. The probe was perpendicular to the muscle fiber, and the speed was set to 0.83 mm/s. The average value of 6 replicates for each sample was calculated and recorded as the shear force value.

#### 2.4.4. Electronic Tongue Analysis

The 15 g thawed breast muscle sample was sealed in a polyethylene plastic cooking bag, and then heated in 80 °C water until the center of the meat reached 70 °C. After grinding and homogenizing the meat sample in liquid nitrogen, 5 g of the meat sample was weighed and placed in a 50 mL centrifuge tube. Ultrapure water (20 mL preheated to 38 °C) was added to the tube. A vortex mixer was used to vigorously mix the contents for 30 min. Then, another 20 mL of ultrapure water (preheated to 38 °C) was added, and the mixture was subjected to ultrasound at 37 °C for 30 min. Following the ultrasound treatment, the tube was centrifuged at 5000 rpm in a room temperature environment for 10 min. The supernatant was carefully decanted into a sand core funnel for vacuum filtration. The volume of the filtrate was measured, and it was diluted by a factor of 2 before analysis.

The electronic tongue (TS-sa402b, INSENT Inc., Tokyo, Japan) was used to evaluate the taste characteristics of the breast muscle sample. The instrument consists of five sensors (CA0 stands for sourness, C00 for bitterness and its aftertaste, AE1 for astringency and its aftertaste, AAE for umami and its richness, CT0 for saltiness and GL1 for sweetness). The sensor was soaked with a reference solution (30 mM KCl + 0.3 mM tartaric acid) for 24 h before the measurement. The sensor was washed in the cleaning solution for 90 s and in the reference solution for 120 s + 120 s, and soaked in the sample solution for 60 s. The five basic tastes were measured four times, and data from the last three times were used for statistical analyses.

#### 2.4.5. Volatile Flavor Profile Analysis

The analysis was conducted using a Q Exactive Orbitrap mass analyzer equipped with a TriPlus RSH autosampler (Thermo Fisher Scientific, Berlin, Germany) and Trace 1310 GC (Thermo Fisher Scientific, Germany). The headspace solid-phase microextraction procedure was selected and performed in a TriPlus RSH autosampler.

After grinding and homogenizing the breast muscle sample in liquid nitrogen, we weighed 2 g of meat sample and placed it in a headspace vial. We added 5 μL of internal standard solution (2-methyl-3-heptanone solution, concentration 0.05 μg/μL, dissolved in n-hexane) to the vial. Promptly, the vials were sealed using a magnetic cap equipped with a PTFE–silicone septum. They were then heated in a 90 °C water bath for 30 min and permitted to reach room temperature for 1 h. Incubation of the sample was carried out at 55 °C for a duration of 20 min, and extraction was performed under the same temperature conditions for 40 min, utilizing a 50/30 µm DVB/CAR/PDMS fiber (Supelco, Bellefonte, PA, USA). Subsequently, the extraction head was automatically introduced into the injector and desorbed at 250 °C for 3 min. To prevent cross-contamination between successive analyses, the extraction head underwent conditioning at 270 °C for 10 min.

The gas chromatography (GC) column: a VF-WAX ms column (Agilent, USA). Carrier gas: Helium (99.9999%), 1.0 mL/min, split ratio of 5:1. Injector temperature: 250 °C. The ramped temperature program: 40 °C, 2.0 min; 230 °C (4 °C/min), 5 min. Transfer line temperature: 250 °C.

The MS program was as follows. Electron impact ionization: 70 eV, full-scan mode. The resolution: 60,000 full widths at half maximum. The scan range: 30 to 400 *m*/*z*. The automatic-gain-control target: 1 × 10^6^. The ion source temperature: 280 °C. The transfer line temperature: 250 °C.

TraceFinder (V 4.1) was used to process and deconvolute the data. In the course of testing, a blank sample and a quality control sample were introduced at intervals of 10 samples to validate and maintain the stability of the instrument. Volatile compounds were identified by utilizing linear retention indices and mass spectra derived from NIST17 (V 2.3), Wiley9, and authentic flavor standards. The HRF scores > 95. The match factor based on the MS pattern > 750. The difference in the retention index for the domestic library < 20. The difference in the retention index for the NIST library < 50. Volatile compounds were quantified using the internal standard.

### 2.5. Transcriptome Analysis

Using TRIzol reagent, RNA extraction was performed on tissue samples obtained from the breast muscle. The quality and quantity of all extracted RNA were assessed using Nanodrop and agarose gel electrophoresis. PrimerScript RT Master Mix (Takara, Beijing, China) was used for cDNA library preparation. Sequencing on an Illumina X-Ten platform was performed for library preparations, resulting in the generation of 150 bp single-end reads. Trimmomatic (V 0.36) was utilized to eliminate sequencing adaptors and low-complexity reads [18]. Subsequently, the *Gallus gallus* reference genome (GCA_016699485.1) was employed for mapping clean data through TopHat (V 2.0.11) [19]. To obtain read counts for each gene, HTSeq (V 0.6.1) [20] was employed, and counts per million mapped sequence reads for each gene were computed using edgeR (V 3.20.9) [21].

### 2.6. Statistical Analysis

Partial least squares discriminant analysis (PLS-DA) was performed on the MetaboAnalyst 5.0 platform. Differential metabolites, lipids and volatiles were identified based on univariate analysis criteria, specifically with variable importance in projection (VIP) > 1 and *p* value < 0.05. The VIP value was extracted from the results of PLS-DA. Using pheatmap (V1.0.12), a heatmap was generated, and hierarchical cluster analysis of the differential metabolites among samples was conducted. For the mapping of metabolic pathways related to differential metabolites, the Kyoto Encyclopedia of Genes and Genomes (KEGG) database was employed.

SPSS 17.0 was employed for the analysis of meat quality data, and the outcomes were expressed as the mean ± SD. Significance levels were established at *p* < 0.05. To assess correlations between differential metabolites and meat quality indices, the Spearman correlation test was executed.

The R package DESeq2 was used to screen differentially expressed genes (DEGs) with |log_2_ (fold change)| ≥ 1 and *p* ≤ 0.05. Utilizing DAVID tools, Gene Ontology (GO) and the KEGG pathway enrichment analyses of DEGs were conducted. The correlations between differential lipids and DEGs were calculated using Spearman’s method. The differential lipid and DEG pairs with r > 0.4 and *p* < 0.05 were screened. Using Cytoscape (V3.8.2), the network of differential lipids and DEGs was constructed.

## 3. Results and Discussion

### 3.1. Identification of Characteristic Bioactive Metabolites

**Figure 1:**
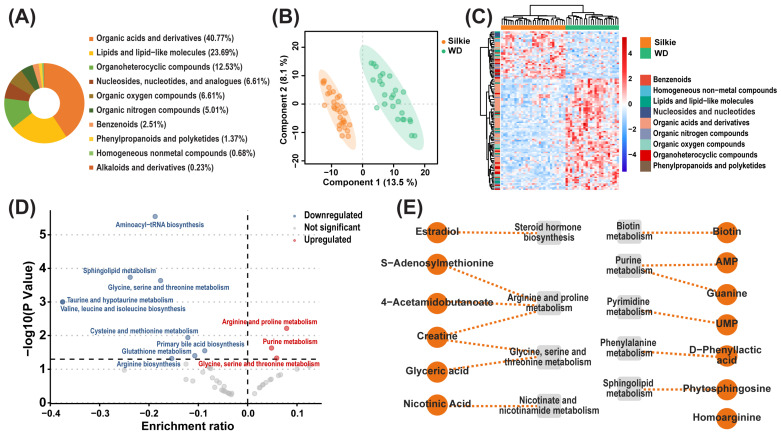
Identification of characteristic bioactive metabolites. (**A**) Classification and percentage of metabolites in the breast muscle of 30 Silkie chickens and 30 Wuding chickens. (**B**) PLS-DA based on overall metabolites. (**C**) Cluster heat map of the differential metabolites. The abscissa represents samples, and the ordinate represents differential metabolites. (**D**) KEGG pathway enrichment analysis of differential metabolites. Each circle shows a corresponding pathway. Red circles represent significantly upregulated pathways, and blue circles represent significantly downregulated pathways. (**E**) Characteristic bioactive metabolites and their related metabolic pathways in Silkie chickens.

A total of 624 metabolites were identified in the breast muscle of 30 Silkie chickens and 30 Wuding chickens. Based on chemical classification, these metabolites mainly included organic acids and derivatives (40.77%) and lipids and lipid-like molecules (23.69%) (Figure 1A). The constructed PLS-DA model successfully distinguished between Silkie and Wuding chickens (Figure 1B), indicating significant differences in metabolite levels between the two breeds. Based on the criteria of VIP > 1 and *p* < 0.05, 38 metabolites were significantly upregulated and 88 metabolites were significantly downregulated in Silkie chickens (Figure 1C, Table S1). KEGG pathway enrichment analysis of these differential metabolites showed that arginine and proline metabolism and purine metabolism were significantly upregulated, while aminoacyl-tRNA biosynthesis, sphingolipid metabolism, and glycine, serine, and threonine metabolism were significantly downregulated in Silkie chickens (Figure 1D).

Thirteen significantly upregulated metabolites with health-promoting effects were identified as characteristic bioactive metabolites of Silkie chickens (Figure 1E). Estradiol plays a role in regulating women’s menstrual cycles [22] and effectively prevents menopausal syndrome [23,24]. These effects are consistent with the traditional Chinese patent medicine “Black Chicken Pills”. *S*-Adenosylmethionine, as a nutritional supplement, has been shown to maintain liver health [25], improve mood [26], and comfort joints [27]. Homo-l-arginine supplementation can attenuate atherosclerosis [28]. Nicotinic acid has pharmacological activities such as vasodilation and blood lipid-lowering effects [29]. Phytosphingosine can exert antidiabetic effects by reducing serum cholesterol levels, reducing free fatty acid levels, improving insulin sensitivity [30,31], and alleviating inflammatory diseases [32]. Glyceric acid can activate mitochondrial metabolism and reduce inflammation in healthy volunteers aged 50–60 years [33,34]. D-Phenyllactic acid can alleviate colitis by regulating microbiota composition, short-chain fatty acid production, and inflammatory responses [35]. Uridine monophosphate (UMP) can enhance cognitive functions [36]. Creatine is the most common dietary supplement with beneficial effects, enhancing exercise performance and protecting nervous and cardiac tissue [37]. Guanine has neurotrophic and neuroprotective effects [38]. 4-acetamidobutanoic acid is strongly correlated with antioxidant activity [39]. Biotin has health benefits [40] and improves skin, hair, and nails [41]. The efficacy of these characteristic bioactive metabolites indicates that Silkie chickens are suitable for the elderly and women to nourish the body.

### 3.2. Identification of Characteristic Bioactive Lipids and Fatty Acids

**Figure 2:**
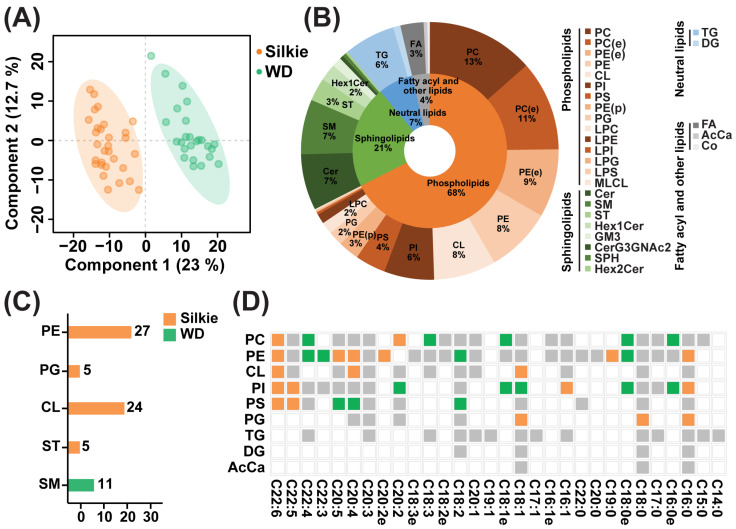
Identification of characteristic bioactive lipids and fatty acids. (**A**) PLS-DA based on overall lipid molecules. (**B**) Distribution of major lipid classes and subclasses. (**C**) The number of differential lipid molecules in each lipid subclass. Orange and green represent lipid molecules that were significantly upregulated and downregulated in Silkie chickens, respectively. (**D**) Heat map of differential fatty acids in Silkie and Wuding chickens. Orange, green, and gray squares indicate fatty acids that were significantly upregulated, significantly downregulated, and not differentially expressed in Silkie chickens, respectively. Transparent squares indicate the absence of this fatty acid.

A total of 653 lipid molecules were identified in the breast muscle of 30 Silkie chickens and 30 Wuding chickens. The PLS-DA model could clearly distinguish between breast muscle samples from the two breeds (Figure 2A). These 653 lipid molecules were categorized into four major classes, which were further subdivided into 28 subclasses. The distribution of the lipid major classes and subclasses is presented in Figure 2B. Phospholipids accounted for the highest proportion (68%), followed by sphingolipids (21%). Based on the criteria of VIP > 1 and *p* < 0.05, differential lipid subclasses between the two breeds were identified. In the breast muscle of Silkie chickens, phosphatidylethanolamine (PE), phosphatidylglycerol (PG), cardiolipin (CL) and sulfatide (ST) were significantly upregulated, and sphingomyelin was significantly downregulated (Table S2). Among them, 27 PE molecules, five PG molecules, 24 CL molecules, and five ST molecules were significantly upregulated, and 11 sphingomyelin molecules were significantly downregulated in Silkie chickens (Figure 2C, Table S3).

To further identify the differential lipid molecules that contributed significantly to the upregulation of CL, PE, PG, and ST, we calculated the relative contribution of each lipid molecule to differences in lipid subclasses. As illustrated in Figure S1A, CL(18:2_18:1_18:1_18:2)-H and CL(18:2_18:1_18:2_18:2)-H contributed to 31% and 25% of the variance in the CL subclass, respectively. As illustrated in Figure S1B, PE(18:0_20:4)-H, PE(16:0_20:4)-H, and PE(36:4)+H accounted for 32%, 13%, and 10% of the variance in the PE subclass, respectively. As illustrated in Figure S1C, PG(16:0_18:1)-H and PG(18:0_18:1)-H contributed 69% and 23% of the variance in the PG subclass, respectively. As illustrated in Figure S1D, ST(m38:0)-H accounted for 77% of the variance in the ST subclass. These eight lipid molecules play an important role in the formation of four characteristic bioactive lipids in Silkie chickens. PE is a lecithin with antioxidant and antiaging effects [42]. CL is a mitochondria-exclusive phospholipid. A reduction in CL content is related to aging and several neurodegenerative diseases, such as Alzheimer’s disease, Parkinson’s disease, and traumatic brain injury [43]. ST is a multifunctional molecule, and ST deficiency is an important contributor to and driver of neuroinflammation and mild cognitive impairment in Alzheimer’s disease [44].

The fatty acid composition is shown in Figure 2D. A total of 116 fatty acids were detected. Based on the criteria of VIP > 1 and *p* < 0.05, 19 fatty acids were significantly upregulated and 17 fatty acids were significantly downregulated in the breast muscle of Silkie chickens (Table S4). Among monounsaturated fatty acids, palmitoleic acid was significantly upregulated in Silkie chickens. Among polyunsaturated fatty acids, DPA and DHA were generally significantly upregulated in Silkie chickens. Palmitoleic acid can significantly ameliorate or prevent insulin resistance and diabetes [45]. DPA and DHA exert myriad health benefits on cardiovascular disease, diabetes, cancer, depression, and cognitive decline [46]. DHA has unique actions in promoting normal brain function [47]. These characteristic lipids and fatty acids play important roles in resisting aging and improving cognition, suggesting that Silkie chicken is beneficial to the elderly and children.

### 3.3. Effects of Characteristic Bioactive Metabolites on Meat Quality Traits

The meat quality traits of 30 Silkie chickens and 30 Wuding chickens are shown in Table 1. In Silkie chickens, the values of pH_24h_ and shear force were higher (*p* < 0.05), and the pressing loss, cooking loss, and meat color L* and b* values were lower (*p* < 0.05). The lower pressing loss and cooking loss indicate that their water-holding capacity was better. Electronic tongue analysis showed that the sensory values of umami, richness, and bitterness were higher than the tasteless point (0) in both Silkie and Wuding chickens, indicating that they could be detected by taste buds. The umami value in the breast muscle of Silkie chickens was higher (*p* < 0.05, Table 2).

**Figure 3:**
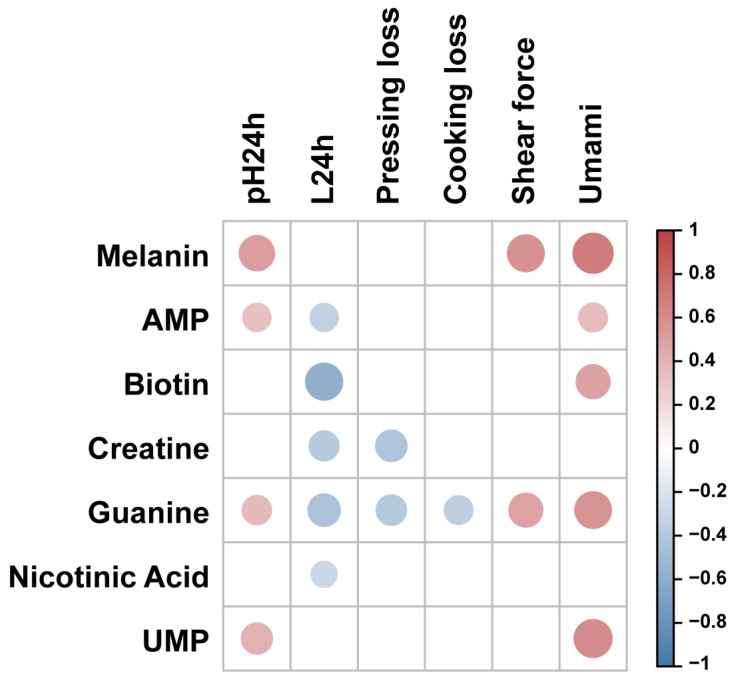
Correlation analysis between characteristic bioactive compounds and meat quality in the breast muscle of Silkie chickens. Red circles represent a significant positive correlation (*r* > 0.3 and *p* < 0.05) and blue circles represent a significant negative correlation (*r* < −0.3 and *p* < 0.05).

We explored the effects of characteristic bioactive metabolites on meat quality traits in Silkie chickens (Figure 3 and Table S5). Correlation analysis showed that melanin was positively correlated with shear force, which may be due to the high melanin content in collagen of Silkie chickens, increasing the toughness of collagen [48] and the shear force [49]. Adenosine 5′-monophosphate (AMP) showed a positive correlation with pH_24h_ and a negative correlation with the L* value. One study showed that the pH value of duck breast muscle was significantly increased (*p* < 0.05) and the L* value was significantly decreased (*p* < 0.05) after AMP treatment [50]. Biotin content was negatively correlated with the L* value. One study showed that addition of biotin to the diet resulted in a lower L* value of pig loin muscle [51]. Creatine showed negative correlations with the L* value and pressing loss. Previous studies have showed that increased the creatine content in muscle could postpone the pH decline after slaughter and improve the water-holding capacity [52,53]. The nicotinic acid content was negatively correlated with the L* value. One study showed that dietary nicotinic acid supplementation linearly decreased (*p* < 0.05) the L* value in the breast muscle of Wulong geese [54]. Umami taste showed positive correlations with AMP, guanine, and UMP levels. Previous studies showed that AMP [55], guanine [56], and UMP [57] could all enhance the umami taste of meat. Therefore, the characteristic bioactive metabolites in Silkie chickens not only exhibited health-related functions, but also improved water-holding capacity and umami taste in breast muscle.

### 3.4. Effects of Characteristic Bioactive Metabolites, Lipids, and Fatty Acids on Characteristic Volatiles

**Figure 4:**
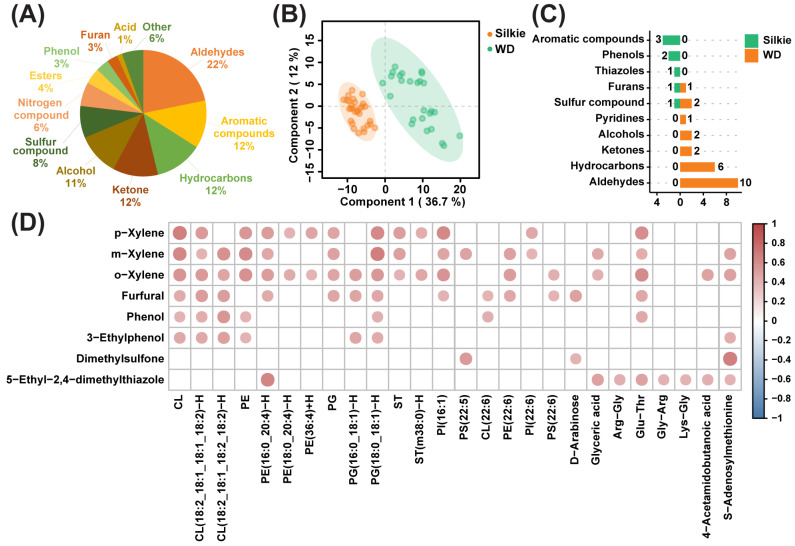
Effects of characteristic bioactive compounds on meat flavor. (**A**) Classification and percentage of volatile flavor compounds. (**B**) PLS-DA based on overall volatile flavor compounds. (**C**) The number of differential volatile flavor compounds in each class. Orange and green represent volatile flavor compounds that were significantly upregulated and downregulated in Silkie chickens, respectively. (**D**) Correlation analysis of eight upregulated volatiles and characteristic bioactive compounds. Red circles indicate a significant positive correlation (r > 0.4 and *p* < 0.05).

A total of 147 volatile flavor compounds were identified and semi-quantified from the breast muscles of 30 Silkie chickens and 30 Wuding chickens. Figure 4A presents the categories and proportions of volatile flavor compounds, with aldehydes accounting for the highest proportion. The PLS-DA model clearly distinguished between Silkie and Wuding chickens based on these 147 volatile flavor compounds (Figure 4B). Based on the criteria of VIP > 1 and *p* < 0.05, eight volatile flavor compounds were significantly upregulated and 24 volatile flavor compounds were significantly downregulated in Silkie chickens (Figure 4C and Table S6). It is worth noting that the upregulated volatiles were mainly aromatic compounds and phenols and the downregulated volatiles were mainly aldehydes and hydrocarbons.

Then, we conducted correlation analysis on eight upregulated volatiles and characteristic bioactive compounds (Figure 4D and Table S7). Furfural was positively correlated with D-arabinose and DHA. Phenol was positively correlated with Glu-Thr. 5-Ethyl-2,4-dimethylthiazole was positively correlated with four dipeptides. Previous studies support these correlations. The main pathways for furfural formation include the Maillard reaction induced by saccharides and nitrogenous compounds [58] and the conversion of polyunsaturated fatty acids [59]. Phenols are mainly derived from amino acid degradation [60]. 5-Ethyl-2,4-dimethylthiazole is mainly formed by nonenzymatic browning reactions between reducing sugars and amino acids [61]. The above results indicated that characteristic bioactive metabolites, lipids, and fatty acids may play important roles in the formation of characteristic volatiles in the breast muscle of Silkie chickens.

### 3.5. Identification of Key DEGs Involved in Synthesis of Characteristic Bioactive Metabolites, Lipids, and Fatty Acids

**Figure 5:**
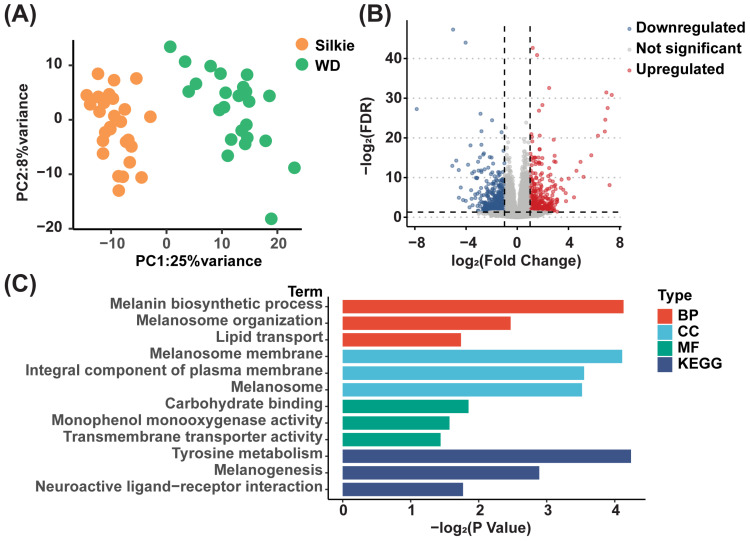
Statistical analysis of DEGs. (**A**) Principal component analysis based on overall transcripts. (**B**) Volcano plot of DEGs in the breast muscle of Silkie and Wuding chickens. (**C**) GO enrichment analysis and KEGG pathway enrichment analysis of upregulated genes in Silkie chickens.

We performed transcriptome sequencing on the breast muscle tissues of 30 Silkie chickens and 30 Wuding chickens, and the two breeds could be well separated based on gene expression levels (Figure 5A). Through further differential analysis, we identified 504 DEGs (fold change ≥ 2 and *p* < 0.05), including 212 upregulated and 292 downregulated genes, in Silkie chickens (Figure 5B and Table S8). The results of our GO enrichment analysis and KEGG pathway enrichment analysis of upregulated genes are shown in Figure 5C. The GO database was used to annotate and enrich DEGs in the biological process, cellular component, and molecular function categories. In the biological process category, we noticed that in addition to the significant upregulation of “melanin biosynthetic process” and “melanosome organization,” “lipid transport” was also significantly upregulated in Silkie chickens.

**Figure 6:**
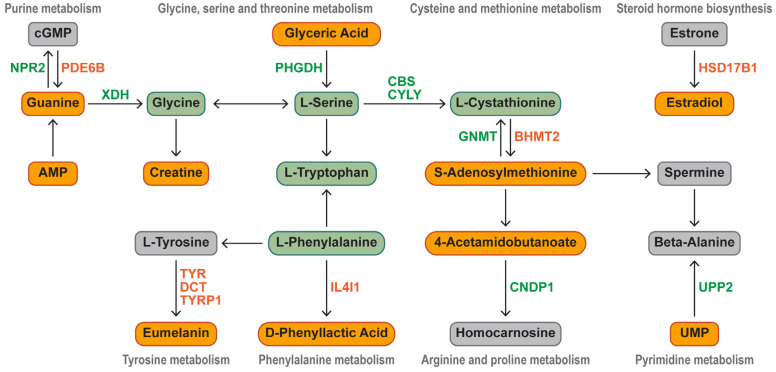
Identification of key DEGs that regulate characteristic metabolic pathways. Orange represents significantly upregulated metabolites and genes in Silkie chickens, and green represents significantly downregulated metabolites and genes.

We present a biochemical pathway map that visually demonstrates how key DEGs regulate the significant upregulation of characteristic metabolites in Silkie chickens (Figure 6). Characteristic bioactive metabolites and 15 DEGs were enriched in five amino acid pathways, two nucleotide pathways, and one lipid pathway. Seven upregulated DEGs and eight downregulated DEGs played key roles in the differential accumulation of characteristic metabolites. *NPR2* (encoding a natriuretic peptide receptor) and *PDE6B* (encoding a phosphodiesterase) were differentially expressed and were responsible for the significant upregulation of guanine. The downregulation of *PHGDH* (encoding a phosphoglycerate dehydrogenase) led to the accumulation of glyceric acid. The upregulation of *BHMT2* (encoding a betaine homocysteine *S*-methyltransferase) promoted *S*-adenosylmethionine synthesis, and the downregulation of *GNMT* (encoding a glycine *N*-methyltransferase) decreased *S*-adenosylmethionine breakdown. Estrone was activated by the significantly upregulated *HSD17B1* (encoding a hydroxysteroid 17-beta dehydrogenase) to form estradiol. *UPP2* (encoding a uridine phosphorylase catalyzing the cleavage of uridine) was downregulated, which led to the accumulation of UMP. *CNDP1* (encoding a carnosine dipeptidase transforming 4-acetamidobutanoate into homocarnosine) was downregulated, which led to an increase in the 4-acetamidobutanoate content. The significant upregulation of *IL4I1* promoted the biosynthesis of D-phenyllactic acid.

**Figure 7:**
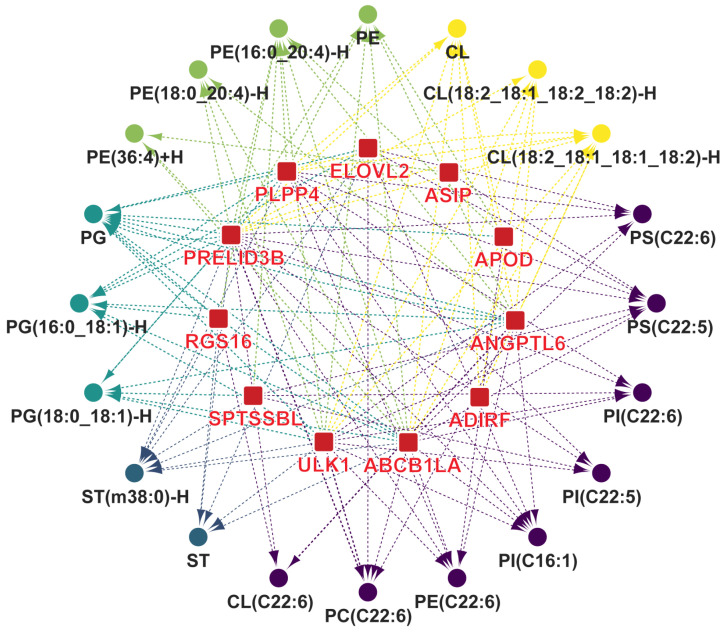
Identification of key DEGs that play a role in the biosynthesis of characteristic lipids and fatty acids. Lines indicate a significant positive correlation (*r* > 0.4 and *p* < 0.05) between gene expression and the content of lipids and fatty acids.

We conducted correlation analysis to identify DEGs that regulate the synthesis of characteristic lipids and fatty acids. A total of 11 lipid metabolism-related DEGs were positively correlated with characteristic lipids and fatty acids (*r* > 0.4 and *p* < 0.05, Figure 7). We identified 11 DEGs that play key roles in the biosynthesis of upregulated characteristic lipids and fatty acids in Silkie chickens. *ADIRF* promotes adipogenic differentiation. *ABCB1LA*, *APOD*, and *PRELID3B* encode proteins that are involved in lipid transport. *ABCB1LA* encodes a protein that is capable of transporting PE [62] and is positively correlated with PE, PE(16:0_20:4)-H, PE(18:0_20:4)-H, and PE(36:4)+H. *APOD* encodes the most abundant and versatile apolipoprotein that facilitates lipid transport and metabolism [63,64]. *PRELID3B* encodes a protein that is involved in phospholipid transport [65] and is positively correlated with PE, PG, and CL. The proteins encoded by *ASIP*, *ELOVL2*, *PLPP4*, and *SPTSSBL* are directly involved in the formation of lipids and fatty acids. *ASIP* affects the saturation of fatty acids [66] and is positively correlated with PS(C22:5). *ELOVL2* is directly involved in polyunsaturated fatty acid biosynthesis [67] and is positively correlated with PS(C22:5), PS(C22:6), and PC(C22:6). *PLPP4* is involved in the synthesis of PE and is positively correlated with PE and PE(16:0_20:4)-H. *SPTSSBL* encodes a protein that catalyzes the first rate-limiting step in sphingolipid biosynthesis [68] and is positively correlated with ST(m38:0)-H. *ANGPTL6* [69], *RGS16* [70], and *ULK* [71] also play important roles in lipid metabolism.

## 4. Conclusions

In the present study, we identified the 13 characteristic metabolites, including estradiol, nicotinic acid, and creatine, which have health-promoting effects such as regulating menstrual cycles, lowering blood lipid levels, and enhancing cognitive functions, and also improve the water-holding capacity and umami taste in breast muscle. Four characteristic lipid subclasses, including CL, and three characteristic fatty acids, including DHA, have antiaging and cognition-enhancing effects and were significantly correlated with upregulated aromatic compounds and phenols in breast muscle. This study deepened our understanding of the characteristic compounds with high nutritional value in Silkie chickens and provided new insights into why consumers prefer using Silkie chicken to nourish their bodies.

## Figures and Tables

**Table 1:** Meat quality traits in Silkie and Wuding chickens.

	Silkie Chickens	Wuding Chickens	*p* Value
pH_24h_	5.75 ± 0.28 ^a^	5.45 ± 0.29 ^b^	0.01
L*_24h_	48.83 ± 2.16 ^b^	59.40 ± 3.30 ^a^	<0.001
a*_24h_	3.00 ± 1.21	2.57 ± 1.53	
b*_24h_	5.24 ± 1.96 ^b^	8.19 ± 2.92 ^a^	<0.001
Pressing loss (%)	7.30 ± 1.51 ^b^	8.31 ± 1.88 ^a^	0.02
Cooking loss (%)	13.95 ± 1.61 ^b^	15.19 ± 2.22 ^a^	0.02
Shear force (kg)	3.62 ± 0.93 ^a^	3.15 ± 0.58 ^b^	<0.001
Melanin content (ng/g)	439.72 ± 337.85 ^a^	0.00 ± 0.00 ^b^	<0.001

^a,b^ Different letters in the same row indicate significant differences (*p* < 0.05). Data are presented as mean ± SD (*n* = 30).

**Table 2:** Electronic tongue analysis in Silkie and Wuding chickens.

	Silkie Chickens	Wuding Chickens	*p* Value
Umami	8.52 ± 0.51 ^a^	8.02 ± 0.49 ^b^	<0.01
Richness	5.39 ± 0.43	5.32 ± 0.48	
Bitterness	1.90 ± 0.58	1.79 ± 0.58	
Aftertaste-B	−0.26 ± 0.16	−0.33 ± 0.23	
Astringency	−3.97 ± 0.36 ^b^	−3.68 ± 0.32 ^a^	0.01
Aftertaste-A	0.004 ± 0.03	−0.01 ± −0.01	
Saltiness	−12.10 ± 0.50	−12.22 ± 0.52	
Sourness	−21.73 ± 1.78 ^b^	−19.90 ± 1.66 ^a^	<0.01

^a,b^ Different letters in the same row indicate significant differences (*p* < 0.05). Data are presented as mean ± SD (*n* = 30).

## Data Availability

The datasets presented in this study can be found in CNCB repositories. The accession numbers can be found in BioProject CRA012494 (https://ngdc.cncb.ac.cn/gsub/submit/gsa/subCRA019702/finishedOverview).

## Supplementary Materials

The following supporting information can be downloaded at: https://www.mdpi.com/article/10.3390/foods13060969/s1. Table S1: Differential metabolites in breast muscle between Silkie and Wuding chickens. Table S2: Differential lipid subclasses in breast muscle between Silkie and Wuding chickens. Table S3: Differential lipid molecules in breast muscle between Silkie and Wuding chickens. Table S4: Differential fatty acids in breast muscle between Silkie and Wuding chickens. Table S5: Correlation between characteristic metabolites and meat quality traits. Table S6: Differential volatile flavor compounds in breast muscle between Silkie and Wuding chickens. Table S7: Correlation between characteristic metabolites, lipids, and volatiles. Table S8: DEGs in breast muscle between Silkie and Wuding chickens. Figure S1: Contribution of significantly upregulated lipid molecules to significantly upregulated lipid subclasses in Silkie chickens. The bar graphs show the proportion of differential lipid molecules in each lipid subclass. The pie chart shows the difference in lipid molecules as a percentage of the difference in the lipid subclass.
