# Supplementary material for: Identification of Characteristic Bioactive Compounds in Silkie Chickens, Their Effects on Meat Quality, and Their Gene Regulatory Network
Source: Foods. 2024 Mar 21;13(6):969. doi: 10.3390/foods13060969 (PMC10970468; doi:10.3390/foods13060969)
Supplement: Supplementary file 1 [file foods-13-00969-s001.zip › Supplemental files/Figure S1.pdf]

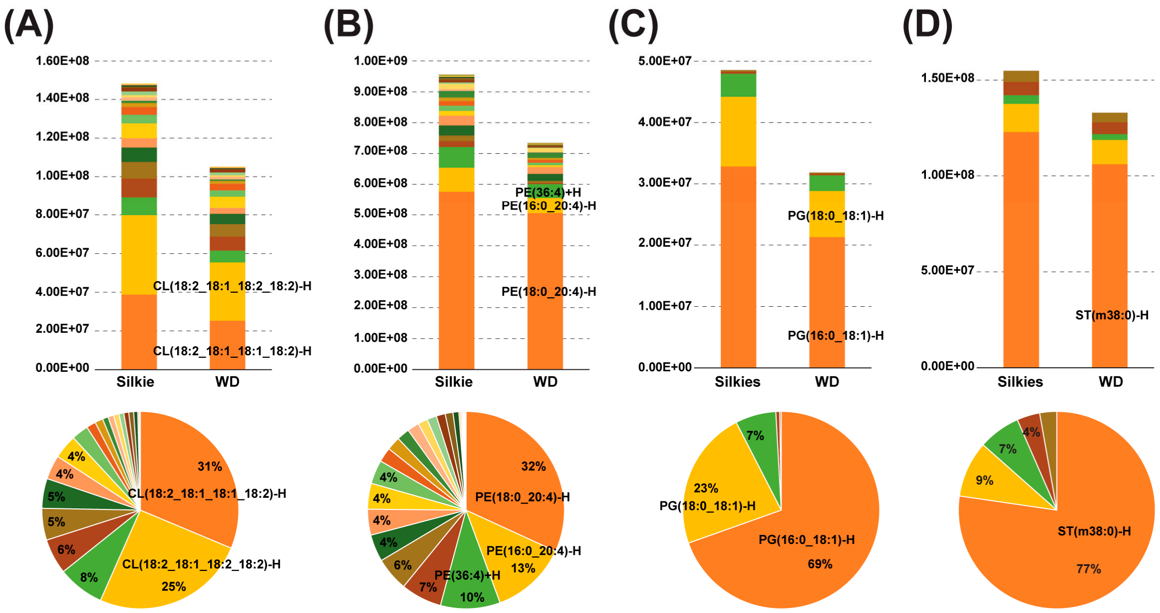

Figure S1. Contribution of significantly upregulated lipid molecules to significantly upregulated lipid subclasses in Silkie chickens.
